# Risk factors of under-five mortality in Ethiopia using count data regression models, 2021

**DOI:** 10.1016/j.amsu.2022.104764

**Published:** 2022-09-22

**Authors:** Alemayehu Siffir Argawu, Gizachew Gobebo Mekebo

**Affiliations:** Department of Statistics, Ambo University, Ambo, Regional State of Oromia, Ethiopia

**Keywords:** Under-five mortality, Risk factors, Zero-inflated Poisson model, Ethiopia

## Abstract

**Background:**

Despite the global reduction in under-five mortality, still many children die before their fifth birthday in Ethiopia. The main aim of this study was to identify determinants of under-five mortality using count data regression models based on 2019 Ethiopia mini demography and health survey data.

**Methods:**

The data source for this study was the 2019 Ethiopia mini demography and health survey data. Various count data regression models were applied to identify the determinants of under-five mortality.

**Results:**

A total of 5,535 mothers with children aged 0–59 months were included in the study. Of the total, 1,277 (23.07%) women had lost at least one child by death before celebrating fifth birthday. Zero-Inflated Poisson model was found to be the best model, and it revealed that mother's age, marital status, mother's age at 1st birth, place of delivery, current contraceptive type used, type of cooking fuel, residence, region, religion, time to get drinking water, number of children at home, birth order, and birth type were significant factors that determine U5 mortality in Ethiopia.

Mothers aged 15–24 years (IRR = 1.24, p = 0.007) and above 24 years (IRR = 1.66, p = 0.000) at their 1st births, mothers from rural area (IRR = 1.27, p = 0.000), mothers traveled for 1–30 min (IRR = 1.62, p = 0.000) and >30 min (IRR = 1.82, p = 0.000) to get drinking water, mothers used charcoal (IRR = 1.86, p = 0.009) and wood (IRR = 1.64, p = 0.033), children with birth order of 2nd-3rd (IRR = 3.91, p = 0.000), 4th -5th (IRR = 13.14, p = 0.000), 5th and above (IRR = 38.17, p = 0.000), and multiple born children (IRR = 1.5, p = 0.000) had higher risk of under-five mortality while mothers aged 25–34 years (IRR = 0.73, p = 0.000), unmarried mothers (IRR = 0.68, p = 0.004), mothers delivered in public health sectors (IRR = 0.59, p = 0.000), mothers used Pill/IUD (IRR = 0.64, p = 0.018), mothers who had 3 to 5 (IRR = 0.51, p = 0.000) and more than 5 (IRR = 0.27, p = 0.000) children at home had lower risk of under-five mortality.

**Conclusions:**

Mothers should be encouraged to deliver at health institutions. Mothers also should be given awareness to use Pill/IUD contraceptive type. Moreover, facilitating rural areas to get electricity and drinking water near to homes helps to reduce the burden of U5M and to be in line with sustainable development goal.

## Highlights

• Our study was an attempt to identify the risk factors for under-five mortality in Ethiopia.
• We used 2019 Ethiopian Mini Demographic and health Survey data.
• Analyses were done using Statistical sofwares R, Stata, and SPSS.
• Count Data Regression Models were employed to identify the risk factors of under-five mortality.
• The best model that fits the data well was selected using selection criterion like AIC, BIC.
• Zero-Inflated Poisson model was found to fit the data well.
• The study found that mother's age, marital status of mother, age of mother's at first birth, place of delivery, place of residence, time to get drinking water, number of children at home, birth order, type of birth were statistically significant determinants for under-five mortality in Ethiopia.

## 1 Background

The ongoing child mortality reduction is considered as one of the most critical successes in public and population health of the past three decades. The deaths of under-five children have fallen from 12.5 million in 1990 to 5.3 million in 2018 [1]. Nevertheless this progress, there is still a heavy burden of child deaths due to preventable causes (like pneumonia, malaria, and diarrhea). This burden has both social and economic consequences. In the WHO African region alone, the cost of child mortality amounted to 150.3 billion US dollars in 2013 [2].

In 2019, nearly half (49%) of all U5 deaths occurred in just five countries: Nigeria, India, Pakistan, the Democratic Republic of the Congo and Ethiopia. In 2019, sub-Saharan Africa had an average U5M rate of 76 deaths per 1,000 live births. That is equivalent to 1 child in 13 dying before reaching age 5. This rate is 20 times higher than that of 1 in 264 in the region of Australia and New Zealand [3].

In Ethiopia, as the 2019 EMDHS report shows the trends of U5M declined from 123 deaths per 1,000 live births in 2005 to 59 deaths per 1,000 live births in 2019, a 52% decrease [4]. Even if the U5M has decreased in Ethiopia, it is not as fast as needed and insufficient to reach the Growth and Transformation Plan and Sustainable Development Goal in the country. Despite this reduction, still many children are dying before their fifth birthdays in Ethiopia.

Understanding determinants of under-five mortality is essential to inform public health policies and design strategies to accelerate the reduction of under-five mortality [5,6]. In Ethiopia, many researchers had used the 2016 EDHS data to examine risk factors of U5M using different models [[7], [8], [9], [10]]. However, they did not include some important variables like time to get drinking water, age of household head, number of household members, relationship to household head, type of cooking fuel, number of 5 and U5 children, and number of children at home. But, our study included these variables in addition to others. Thus, this study aimed to identify risk factors of under-five mortality using count data regression model based on 2019 Ethiopia mini demography and health survey data.

## 2 Methods

### 2.1 Population and sample design

The study was a retrospective design study, and the data source was the 2019 Ethiopia mini demography and health survey data. The census frame was a complete list of 149,093 enumeration areas (EAs), among them, 35,292 are in urban areas and 113,801 in rural areas. In the first stage, 305 EAs (93 in urban areas and 212 in rural areas) were selected with probability proportional to EAs size and the household listing was carried out in each EA. In the second stage, 30 households per cluster were selected with equal probability selection [4].

### 2.2 Sample in the study

A total of 8,663 households were successfully interviewed with a response rate of 99%. In the interviewed households, 9,012 women aged 15–49 were identified for individual interviews [4]. A total of 5,535 women with children aged 0–59 months from the data were included in this study.

### 2.3 Variables in the study

The outcome variable was total number of children who died under the age of 5 per woman in her lifetime measured as count 0, 1, 2, … The predictor variables in this study were mother's age, mother's education level, mother's literateness, marital status, religion, mother's age at 1st birth, place of delivery, current contraceptive type, residence, region, number of women in the home, source of water, toilet facility, time to get drinking water, age of household head, wealth index, number of household members, relationship to household head, type of cooking fuel, number of U5 children, number of children at home, birth order, birth type, and child's sex.

### 2.4 Method of data analysis

The under-five mortality data experienced excess zeros characterized by over-dispersion and heteroscedasticity. The most popular distribution for modeling such data is zero-inflated model and hurdle models. The over-dispersion has been explained as heterogeneity that has not been accounted for unobserved population which consists of several sub-populations in this case of Poisson type, but the sub-population membership is not observed in the sample. This excess variation may be occurred incorrect inference about parameter estimates, standard errors, tests, and confidence intervals. The Negative binomial model addresses the issue of over-dispersion by including a dispersion parameter to accommodate the unobserved heterogeneity in the count data. However, it cannot address the over-dispersion caused by an excessive number of zeros, in such case zero-inflated and Hurdle models are appropriate. Zero-inflated models mix a count component and a point mass at zero, allowing for over-dispersion [11,12].

The likelihood-ratio test is used to test the null hypothesis of no over-dispersion (i.e., the Poisson model is preferred) against the alternative hypothesis the over-dispersion parameter is different from zero (i.e., the data would be better fitted by the negative binomial regression). Furthermore, log likelihood, MSE, MAE, AIC and BIC were used to compare various candidate models, and the model with the smallest AIC and BIC value was considered as a better fit [13]. The data analysis was done by using SPSS 25, STATA 14, and R 4.1.0 versions software packages.

## 3 Results

### 3.1 Descriptive statistics

A total of 5,535 women were included of which 1,277 (23.07%) women had lost at least one child by death before celebrating fifth birthday whereas the remaining 4,258 (76.93%) of the mothers had not lost their U5 children by death. This indicates zero outcomes were large in number. The histograms are highly picked at the beginning (the zero values). However, large number of under-five deaths per mother were observed less frequently. Additional screening of number of child deaths showed that the variance (0.57) was greater than the mean (0.35) indicating over-dispersion (Table 1).

**Table 1:** Number of U5 deaths per mother in 2019 EMDHS.

Number of deaths	Frequency	Percent
0	4,258	76.93
1	836	15.10
2	305	5.51
3	99	1.79
4	21	0.38
5	6	0.11
6	8	0.14
7	2	0.04
Total	5,535	100
Mean	0.35	
Variance	0.57	

### 3.2 Models comparisons criteria

**Fig. 1:**
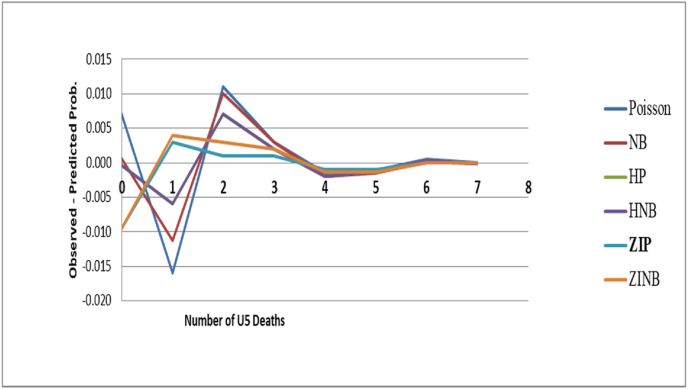
Plots of observed minus predicted probability of number of U5 deaths for six models.

At the point when the significant wellspring of over-dispersion is a dominance of zero tallies, the subsequent over-dispersion cannot be modeled precisely with the negative binomial regression model. An elective path for demonstrating this kind of data is the zero-inflated Poisson or zero-inflated negative binomial regression model which considers the excess of zeroes. And, the overall models comparison was presented in Table 2. The minimum BIC was observed for the NB model, followed by Poisson and ZIP models. However, other validity indices of the model (maximum log likelihood and minimum MSE and MAE) favored for ZIP and ZINB models over all other models. But, the ZIP model is more preferable than ZINB by minimum AIC. In addition, the plot of observed minus predicted probability of the number of U5 deaths at each count was displayed in Fig. 1. The line of difference between observed minus predicted probability of number of U5 deaths was close to the reference zero line, showing the data is better fit of ZIP model than ZINB and other models.

**Table 2:** Overall models comparison by model fit characteristics.

Test statistics	Model					
	Poisson	NB	HP	HNB	ZIP	ZINB
Observed 0 value	4258	4258	4258	4258	4258	4258
Predicted 0 value	4214	4250	4258	4258	4307	4307
Log likelihood	−3343.9	−3337.4	−3154.8	−3154.8	−3115.9	−3115.9
AIC	6799.9	6788.8	6533.6	6535.6	**6455.8**	6457.8
BIC	7170.6	7166.1	7274.8	7283.5	7197.2	7205.8
MSE	5.377	5.445	0.394	0.394	0.375	0.375
MAE	2.083	2.095	0.365	0.365	0.358	0.358

### 3.3 Mothers socio-demography determinants in the fitted model

The rate of non-zero U5 death for children born to 25–34 years old mother decreased by 27% (IRR = 0.73, 95% CI: 0.58–0.89) as compared to 15–24 years old mother keeping other variables held constant in the model. Concerning mother's marital status, the risk of U5M for unmarried mothers was 0.68 (IRR = 0.68, 95% CI: 0.5–0.86) times lower among U5 children born to married mothers. With regard to mother's religion, the risk of U5M for children whose mothers who follow Muslim, protestant and other religions were 1.35 (IRR = 1.35, 95% CI: 1.18–1.51), 1.18 (IRR = 1.18, 95% CI: 1.00–1.35) and 1.69 (IRR = 1.69, 95% CI: 1.16–2.23) times higher as compared to children whose mothers who follow Orthodox religion, respectively. Similarly, the rates of U5 death for children whose mothers were 15–24 and above 24 years old increased by 24% (IRR = 1.24, 95% CI: 1.05–1.44) and 66% (IRR = 1.66, 95% CI: 1.3–2.03) as compared to mothers younger than 15 years, respectively. Regarding place of delivery, mother who delivered in public health sector had 0.59 (IRR = 0.59, 95% CI: 0.52–0.66) times lower risk U5 death rate as compared to mother who delivered in home. Similarly, the incidence rate of U5 death for mothers who used Pill/IUD was decreased by 36% (IRR = 0.64, 95% CI: 0.41–0.88) as compared to mothers who did not use any contraceptive type. In the zero-inflated part, the estimated odds that the number of U5 death becomes zero for mothers who follow Muslim and protestant decreased by 87% (AOR = 0.13, 95% CI: 0.06–0.21) and 45% (AOR = 0.55, 95% CI: 0.18–0.68) as compared to mothers who follow Orthodox religion, respectively (Table 3).

**Table 3:** ZIP regression fitted model for the number of U5 children deaths by mothers’ socio-demographic and related characteristics in Ethiopia, EMDHS, 2019.

Count inflation model coefficients								
Variable	Category	Estimate	SE	Z-value	P-value	IRR	95% CI for IRR	
Intercept	Constant	−3.29	0.37	−9.0	0.000	0.04	0.01	0.06
Mother's education level (Ref: No education)	Primary	−0.11	0.06	−1.96	0.05	0.90	0.79	1.00
	Secondary	−0.08	0.12	−0.66	0.512	0.93	0.72	1.14
	Higher	−0.12	0.15	−0.82	0.410	0.89	0.63	1.14
Mother's age (Ref: 15–24 years)	25–34 years	−0.31	0.11	−2.87	0.004	0.73	0.58	0.89
	35–49 years	−0.05	0.13	−0.4	0.692	0.95	0.71	1.19
Marital status (Ref: Married)	Unmarried	−0.38	0.14	−2.82	0.005	0.68	0.50	0.86
Mother's religion (Ref: Orthodox)	Muslim	0.3	0.06	4.7	0.000	1.35	1.18	1.51
	Protestant	0.16	0.08	2.16	0.031	1.18	1.00	1.35
	Other	0.53	0.16	3.26	0.001	1.69	1.16	2.23
Literacy (Can't read sentence)	Read sentence	0.02	0.08	0.26	0.795	1.02	0.87	1.17
Mother's age at 1st birth (Ref: < 15 years)	15–24 years	0.22	0.08	2.7	0.007	1.24	1.05	1.44
	Above 24 years	0.51	0.11	4.59	0.000	1.66	1.30	2.03
Place of delivery (Ref: Home)	Public sector	−0.53	0.06	−8.73	0.000	0.59	0.52	0.66
	Private sector	−0.14	0.09	−1.47	0.143	0.87	0.71	1.03
Current contraceptive used type (Ref: Not using)	Pill/IUD	−0.44	0.19	−2.37	0.018	0.64	0.41	0.88
	Injections	−0.09	0.08	−1.1	0.270	0.91	0.76	1.06
	Implants/Norplant	0.16	0.1	1.68	0.093	1.18	0.95	1.4
	Other	0.13	0.18	0.70	0.487	1.14	0.73	1.54
**Zero inflation model coefficients**								
Variable	Category	Estimate	SE	Z-Value	P-value	AOR	95% CI for AOR	
Intercept	Constant	0.12	2.15	0.06	0.954	1.13	−3.7	5.91
Mother's education. level (Ref: No education)	Primary	0.05	0.28	0.16	0.871	1.05	0.47	1.63
	Secondary	0.44	0.49	0.9	0.368	1.55	0.07	3.04
	Higher	0.79	0.66	1.2	0.23	2.2	−0.6	5.03
Mother's age (Ref: 15–24 years)	25–34 years	0	0.42	0.01	0.995	1.00	0.18	1.83
	35–49 years	0.59	0.57	1.03	0.302	1.8	−0.2	3.83
Marital status (Ref: Married)	Unmarried	0.32	0.61	0.53	0.598	1.38	−0.3	3.05
Mother's religion (Ref: Orthodox)	Muslim	−2.02	0.29	−6.84	0.000	0.13	0.06	0.21
	Protestant	−0.6	0.12	−2.24	0.000	0.55	0.18	0.68
	Other	−1.59	0.83	−1.91	0.057	0.20	−0.1	0.54
Literacy (Can't read sentence)	Read sentence	0.16	0.37	0.42	0.676	1.17	0.31	2.03
Age of mother at 1st birth (Ref: < 15 years)	15–24 years	−0.64	0.22	−2.99	0.003	0.53	0.30	0.75
	Above 24 years	−1.91	0.52	−3.66	0.000	0.15	0.00	0.3
Place of delivery (Ref: Home)	Public sector	−11.93	6.52	−4.12	0.125	0.00	0.00	0.00
	Private sector	−20.42	301.02	−0.07	0.946	0.00	0.00	0.00
Current contraceptive type (Ref: Not using)	Pill/IUD	−2.4	1.52	−1.58	0.114	0.09	−0.2	0.36
	Injections	−0.15	0.39	−0.4	0.692	0.86	0.2	1.51
	Implants/Norplant	0.53	0.56	0.93	0.351	1.69	−0.2	3.56
	Other	1.9	0.86	2.21	0.027	6.69	−4.6	18

### 3.4 Mother's household head related determinants in the fitted model

The risk of U5M among children of mothers from rural area was increased by 27% (IRR = 1.27, 95% CI: 1.06–2.24) compared to urban area mothers. Mothers who living in Addis Ababa city decreases the incidence of U5M by 62% (IRR = 0.38, 95% CI: 0.13–0.63) compared to those living in Tigray region. The risks of U5M were increased by 78%, 64%, 51% and 35% for mothers living in Afar, Somali, Oromia and Benishangul-Gumuz regions compared to those living in Tigray region, respectively. The incidence rates of U5 death were increased by 62% (IRR = 1.62, 95% CI: 1.27–1.98) and 82% (IRR = 1.82, 95% CI: 1.42–2.22) for mothers who traveled for ≤30 min and >30 min to get drinking water as compared to mothers who get drinking water near to their homes (or took 0 min), respectively. Likewise, the incidence rates of U5 death were increased by 83%, 73% and 57% for children of mothers whose household heads ages were 25–34, 35–44, and above 44 years compared to 15–24 years old household head, respectively. The richest mothers had lower U5M rate (IRR = 0.84, 95% CI: 0.71–0.98) compared to poor mothers. Mothers from more than six household members had lower U5Mrate (IRR = 0.55, 95% CI: 0.37–0.73) compared to mothers from 1 to 3 household members. Regarding mothers’ cooking fuel types, incidence of U5M rates were increased by 86%, 64% and 114% for children of mothers who used charcoal, wood and other cooking fuel types compared to children of mothers used electricity fuel type, respectively (Table 4).

**Table 4:** ZIP regression fitted model for the number of U5 children deaths by mothers’ household related characteristics in Ethiopia, EMDHS, 2019.

Count inflation model coefficients								
Variable	Category	Estimate	SE	Z-value	P-value	IRR	95% CI for IRR	
Residence (Ref: Urban)	Rural	0.24	0.08	1.68	0.000	1.27	1.06	2.24
Region (Ref: Tigray)	Afar	0.58	0.15	3.85	0.000	1.78	1.26	2.31
	Amhara	0.23	0.15	1.55	0.122	1.26	0.89	1.62
	Oromia	0.41	0.14	2.96	0.003	1.51	1.10	1.93
	Somali	0.50	0.15	3.34	0.001	1.64	1.16	2.13
	Benishangul	0.30	0.15	2.00	0.045	1.35	0.95	1.75
	SNNPR	−0.21	0.14	−1.49	0.137	0.81	0.58	1.04
	Gambela	0.30	0.15	1.94	0.053	1.34	0.94	1.74
	Harari	−0.03	0.16	−0.18	0.856	0.97	0.68	1.27
	Dire Dawa	−0.28	0.19	−1.42	0.156	0.76	0.47	1.05
	Addis Ababa	−0.97	0.34	−2.84	0.005	0.38	0.13	0.63
Source of water (Ref: Unimproved)	Improved	0.06	0.06	1.01	0.313	1.06	0.94	1.17
Time to get drinking water (Ref: 0 Minutes)	≤30 min	0.48	0.11	4.32	0.000	1.62	1.27	1.98
	>30 min	0.60	0.11	5.35	0.000	1.82	1.42	2.22
Toilet facility (Ref: Unimproved)	Improved	−0.13	0.09	−1.51	0.130	0.88	0.73	1.03
Age of household head (Ref: 15–24)	25–34	0.60	0.20	3.09	0.002	1.83	1.13	2.53
	35–44	0.55	0.20	2.71	0.007	1.73	1.04	2.41
	Above 44	0.45	0.20	2.21	0.027	1.57	0.94	2.20
Wealth index (Ref: Poor)	Medium	−0.08	0.08	−1.03	0.301	0.92	0.78	1.06
	Rich	−0.17	0.08	−2.09	0.036	0.84	0.71	0.98
Number of household members (Ref:1–3)	4–6	−0.20	0.15	−1.31	0.190	0.82	0.58	1.06
	Above 6	−0.59	0.17	−3.55	0.000	0.55	0.37	0.73
Num. of. Women in household (Ref: One)	Above one	0.09	0.20	0.47	0.639	1.10	0.67	1.52
Relationship to household head (Ref: Head)	Wife/husband	0.03	0.09	0.34	0.733	1.03	0.85	1.21
	Other	0.50	0.14	3.59	0.000	1.64	1.20	2.08
Type of cooking fuel (Ref: Electricity)	Charcoal	0.62	0.24	2.63	0.009	1.86	1.00	2.71
	Wood	0.50	0.23	2.13	0.033	1.64	0.89	2.39
	Other	0.76	0.25	2.98	0.003	2.14	1.07	3.20
**Zero inflation model coefficients**								
Variable	Category	Estimate	SE	Z-value	P-value	AOR	95% CI for AOR	
Residence (Ref: Urban.)	Rural	0.45	0.31	2.71	0.000	1.32	1.12	2.48
Region (Ref: Tigray)	Afar	3.17	0.72	4.41	0.000	23.70	−9.68	57.09
	Amhara	1.57	0.73	2.15	0.032	4.78	−2.05	11.62
	Oromia	0.83	0.64	1.29	0.196	2.30	−0.61	5.21
	Somali	1.65	0.66	2.49	0.013	5.19	−1.54	11.92
	Benishangul-Gumuz	0.05	0.70	0.07	0.947	1.05	−0.39	2.49
	SNNPR	−1.77	0.85	−2.09	0.037	0.17	−0.11	0.45
	Gambela	−0.53	0.70	−0.75	0.452	0.59	−0.22	1.40
	Harari	−12.21	63.90	−0.19	0.848	0.00	0.00	0.00
	Dire Dawa	−22.05	859.05	−0.03	0.980	0.00	0.00	0.00
	Addis Ababa	−2.91	3.73	−0.78	0.436	0.05	−0.35	0.45
Source of drinking water (Ref: Unimproved)	Improved	0.16	0.25	0.65	0.517	1.18	0.60	1.76
Time to get water source (Ref: 0 Minute)	≤30 min	0.18	0.17	1.09	0.276	1.20	0.81	1.59
	>30 min.	−0.14	0.12	0.45	0.210	0.87	0.63	1.11
Toilet facility (Ref: Unimproved)	Improved	−0.51	0.31	−1.68	0.093	0.60	0.24	0.96
Age of household head (Ref: 15–24)	25–34	−0.06	0.52	−0.12	0.908	0.94	−0.02	1.90
	35–44	0.16	0.69	0.23	0.821	1.17	−0.40	2.74
	Above 44	−0.30	0.70	−0.43	0.670	0.74	−0.28	1.76
Wealth index (Ref: Poor)	Medium	−0.21	0.38	−0.56	0.574	0.81	0.21	1.41
	Rich	0.44	0.42	1.05	0.295	1.55	0.28	2.82
Number of household members (Ref:1–3)	4–6	0.64	0.62	1.04	0.297	1.90	−0.39	4.19
	More than 6	0.71	0.63	1.12	0.263	2.03	−0.48	4.54
Number of eligible women in household. (Ref: One)	More than one	3.90	4.28	0.91	0.363	49.24	−364.1	462.2
Relationship to household head (Ref: Head)	Wife/husband	−0.11	0.36	−0.30	0.764	0.90	0.27	1.53
	Other	0.45	0.44	1.03	0.302	1.57	0.22	2.93
Type of cooking fuel (Ref: Electricity)	Charcoal	1.66	1.88	0.88	0.377	5.24	−14.1	24.51
	Wood	2.97	1.93	1.53	0.125	19.41	−54.0	92.95
	Other	1.81	2.03	0.89	0.372	6.11	−18.2	30.36

### 3.5 Child related determinants in the fitted model

The incidence of U5M rate was decreased by 63% (IRR = 0.37, 95% CI: 0.18–0.57) for more than three the number of 5 and under children in the household compared to only one child in the household. Likewise, the incidences of U5M rates were decreased respectively by 49% (IRR = 0.51, 95% CI: 0.41–0.61) and 73% (IRR = 0.27, 95% CI: 0.20–0.33) for three to five, and above five number of children in the homes compared to less than or equal to two home. The death rates of U5 for children with birth orders of 2nd–3rd, 4th–5th, and above 5th were 3.91, 13.14, and 38.17 times higher compared to 1st birth order child, respectively. On the other hand, multiple birth type of child was 50% increased risk of U5M (IRR = 1.5, 95% CI: 1.19–1.81) compared to a single birth type. In the zero-inflated part, the estimated odds that the number of zero U5 death for two children (5 and under) in the household was 4.09 time higher (AOR = 4.09, 95% CI: 1.41–6.77) compared to only one child in the household (Table 5).

**Table 5:** Zero inflated Poisson regression fitted model for the number of U5 children deaths by children characteristics in Ethiopia, EMDHS, 2019.

Count inflation model coefficients								
Variable	Category	Estimate	SE	Z-value	P-value	IRR	95% CI for IRR	
Number of 5 and under children in household (Ref: One)	Two	−0.04	0.06	−0.71	0.478	0.96	0.84	1.07
	Three	−0.35	0.21	−1.64	0.102	0.71	0.41	1.00
	More than three	−0.99	0.27	−3.65	0.000	0.37	0.18	0.57
Number of children at home (Ref: ≤ 2)	3–5	−0.67	0.10	−6.73	0.000	0.51	0.41	0.61
	Above 5	−1.32	0.12	−10.72	0.000	0.27	0.20	0.33
Birth order number (Ref: 1st)	2nd -3rd	1.36	0.16	8.42	0.000	3.91	2.67	5.15
	4th -5th	2.58	0.18	14.24	0.000	13.14	8.48	17.81
	Above 5th	3.64	0.19	19.24	0.000	38.17	24.0	52.33
Birth type (Ref: Single)	Multiple	0.40	0.11	3.80	0.000	1.50	1.19	1.81
**Zero inflation model coefficients**								
Variable	Category	Estimate	SE	Z-value	P-value	AOR	95% CI for AOR	
Number of 5 and under children in household (Ref: One)	Two	1.41	0.33	4.22	0.000	4.09	1.41	6.77
	Three	−3.20	4.33	−0.74	0.460	0.04	−0.3	0.39
	Above 3	−9.09	5.14	−1.77	0.077	0.00	0.00	0.00
Number of children at home (Ref: ≤ 2)	3–5	0.22	0.39	0.57	0.570	1.24	0.30	2.19
	Above 5	1.96	0.61	3.20	0.001	7.09	−1.4	15.60
Birth order number (Ref: 1st)	2nd - 3rd	−0.48	0.56	−0.85	0.393	0.62	−0.1	1.30
	4th -5th	−1.95	0.70	−2.79	0.005	0.14	−0.1	0.34
	Above 5	−4.56	0.79	−5.79	0.000	0.01	0.00	0.03
Birth type (Ref: Single)	Multiple	−1.79	0.77	−2.32	0.020	0.17	−0.1	0.42

## 4 Discussion

In this study 5,535 mothers with children aged 0–59 months were included, of which 1,277 (23.07%) women had lost at least one child by death before celebrating fifth birthday. The study revealed that mother aged 25–34 years had reduced risk of under-five mortality as compared to 15–24 years old mother. This shows that a younger aged mother face higher U5 child mortality risk. This finding is consistent with other studies conducted in Ethiopia [[7], [8], [9], [10]], Kenya [14], Nigeria [[15], [16], [17]], Columbia [18], Pakistan [19], Bangladesh [20,21], Bolivia [22], and India [23]. This might be due to that younger mothers may also not be socially and psychologically mature enough to deal with the requirements of infant and child care, or they may lack the domestic decision-making authority as compared with older mothers [20]. Whereas, this finding is inconsistent with available literature that points to the fact that maternal age is a strong predictor of child survival [21,[23], [24], [25], [26]].

The study also revealed that the incidence rate of under-five mortality among children whose mothers' ages at first birth were 15–24 years and older than 24 years were significantly more than among children whose mother's age at first birth was less than 15 years. This finding agrees with result of [24,26] while it is contradicts with finding of other study [27].

As presented in this finding, unmarried women had lower risk of under-five deaths than married counterpart. This finding is consistent with study conducted in sub-Saharan Africa countries [28]. However, this finding is inconsistent with other studies findings [15,23,28,29].

This study found that children of Muslim, protestant and other religion followed mothers were having higher risk of dying before the age of five years compared to children whose mothers followed orthodox religion. This might be partly due to the fact that Muslim women tend to face oppositions regarding the use of contraceptive methods from their husbands [30,31].

Findings from this study also revealed that place of delivery is another significant determinant factor of under-five mortality. Children born in a healthcare facility that is in the public or private sectors were at lower risk than those born at home. This might be due to the proper health care and attention these facilities provided to them during and after delivery, and this finding which is confirmed by other studies [10,29,31,32].

Findings of this study indicated that lower mortality rate of children dying before age of five was associated with mothers using contraceptive type. Thus, U5 mortality among children from mothers used pill/IUD contraceptive type was significantly less than children from mothers not used any contraceptive type. This result agrees with previous findings [8,15,29].

The incidence of U5 death was higher among children of mothers living in rural area than those living in urban area. Several researchers found similar results showing children from rural area had higher mortality rate than urban area [9,14,27,28,[32], [33], [34], [35]]. The possible reason could be that urban areas are connected with quality health care services, good education and employment opportunities for mothers.

Region was also found to be significant factor determining under-five mortality. Tigray region had lower risk of under-five mortality compared to other regions except Addis Ababa city administration. This finding is in agreement with other studies which found that region is determinant factor of infant and under-five mortality in Ethiopia [5,7,9,27,32], Kenya [36], Nigeria [15,16,37], Mozambique [38], Ghana [23,39], India [40], and Bangladesh [24]. This might be because of difference in basic infrastructure distribution like health coverage and regional variations in economic development among regions. However, one study showed that region was not significantly related with under-five mortality in Ethiopia [41].

Wealth index was another important determinant factor of under-five mortality. The richest women had significant reduction in under-five mortality compared to the poorest women. This result agrees with various findings [14,16,24,26,35,40,42,43], but it disagrees with the finding of study conducted in Nigeria [29]. In Ethiopia, many previous related studies also showed that the variable wealth index was not significantly determined under-five mortality [31,41,44,45].

This study also found that the incidence rate of U5M increased with increased birth order of the child. This is consistent with other previous studies [4,46,47]. Possible reason might be that as birth order increases, intra-familiar competition for foods and other limited resources essential for child's need will be increased. Moreover, children are more prone to receive most impacts of it. Also as birth order increases level of child care reduces since the mother will have more children to care. Whereas, others findings contradicted this idea [16,38,48].

Furthermore, the study found that U5M was significantly determined by the age of household head, number of household members or family size, time to get the source of drinking water, type of cooking fuel, number of children at home, and birth type. Higher U5M rate was associated with older household head. This finding is consistent with the study [49]. The higher U5M rate was associated with longer time to get drinking water. This is finding is consistent with the previous studies [43,49]. The higher U5M rate was also associated with cooking fuel type of charcoal, wood and other cooking fuel types other than electricity fuel type. This is consistent with prior studies [28,33,42,43,50]. Higher U5M rate was also associated with children of multiple birth type, which is consistent with the studies [[8], [9], [10],^32^,^39^,^47^,^51^]. Lower U5M rate was associated with larger family size. This finding is consistent with studies [7,32,48]. The lower U5M rate was also associated with larger number of children at home. This is consistent with studies [15,52].

## 5 Strength and limitations of the study

This study was based on nationally representative data with a large sample size. Moreover, since it is based on the national survey data, the study result has the potential to give insight for ministry of health, policy-makers, and other concerned bodies to design appropriate intervention strategies both at national and regional levels. However, this study had limitations in that the EMDHS is mostly based on respondents' self-report and might have the possibility of recall bias. In addition, some variables like weight of child at birth [47,53,54], maternal anemia [[54], [55], [56]], child's breastfeeding status [7,47,57], diarrhea [48,58,59], pregnancy desire [60,61], mother's employment status [48,62,63] and fathers' educational status [64,65] were not included in the study because of large number of missing values/unavailability in the dataset.

## 6 Conclusion

This study aimed to identify risk factors of under-five mortality using count data regression model based on 2019 Ethiopia mini demography and health survey data. The ZIP regression model was found to be the best and revealed that mother's age, mother's age at 1st birth, marital status, place of delivery, current contraceptive type used, type of cooking fuel, residence, region, religion, time to get drinking water, number of children at home, birth order, and birth type were significant factors that determine U5 mortality. Moreover, mothers traveled for long hours to obtain drinking water, mothers from Afar, Somali, Oromia and Benishangul regions, mothers from rural area, mothers delivered in homes, mothers used charcoal and wood cooking fuels, children of 2nd and above birth orders, and multiple born children were associated with high incidence of U5M. Thus, Mothers should be encouraged to deliver at health institutions. Mothers also should be given awareness to use Pill/IUD contraceptive type. Moreover, facilitating rural areas to obtain electricity and drinking water near to homes helps to reduce the burden of U5M and to be in line with sustainable development goal.

## Ethics approval and consent to participate

The authors are authorized to download Survey data from the Demographic and Health Surveys (DHS) Program. The data is publicly available and has no personal identifiers.

## Sources of funding

No one funded this research.

## Author contributions

Corresponding author (ASA) had designed the manuscript, written the whole manuscript parts, edited and analyzed the data. Author GGM coded, edited and analyzed the data, edited and revised the manuscript. Finally, both authors read and approved the final manuscript.

## Registration of research studies

We used publicly available secondary data in this study and has no personal identifiers.

Name of the registry:

Unique Identifying number or registration ID:

Hyperlink to your specific registration (must be publicly accessible and will be checked):

## Guarantor

Alemayehu Siffir Argawu (Email:alex.siffir@gmail.com) and Gizachew Gobebo Mekebo (gizmake@gmail.com)

## Data availability

All relevant data and material are available to any interested researchers upon reasonable request from corresponding author.

## Declaration of competing interest

Both authors declare that they no conflict of interest.

## Consent

Not Applicable.

## Declaration of competing interest

Both authors have no conflict of interests.

## Abbreviations

AOR: Adjusted odds ratio
AIC: Akaike's information criterion
BIC: Bayesian information criterion
IRR: incidence rate ratio
DHS: Demographic and Heath survey
EDHS: Ethiopian Demographic and Heath survey
EMDHS: Ethiopian Mini Demographic and Heath survey
NB: Negative Binomial
SNNPR: Southern Nations, Nationalities, and Peoples' Region
U5: Under-five
U5M: Under-five mortality
WHO: World Health Organization
ZIP: Zero-Inflated Poisson
ZINB: Zero-Inflated Negative Binomial.
